# Clinical efficacy of tiotropium in children with asthma

**DOI:** 10.12669/pjms.322.8836

**Published:** 2016

**Authors:** Juan Huang, Ying Chen, Zhen Long, Xiaoqin Zhou, Junhua Shu

**Affiliations:** 1 Juan Huang, Department of Pediatric Internal Medicine, Hubei Maternal and Child Health Care Hospital, Wuhan 430072, China; 2 Ying Chen, Department of Pediatric Internal Medicine, Hubei Maternal and Child Health Care Hospital, Wuhan 430072, China; 3 Zhen Long, Department of Pediatric Internal Medicine, Hubei Maternal and Child Health Care Hospital, Wuhan 430072, China; 4 Xiaoqin Zhou, Department of Pediatric Internal Medicine, Hubei Maternal and Child Health Care Hospital, Wuhan 430072, China; 5 Junhua Shu, Department of Pediatric Internal Medicine, Hubei Maternal and Child Health Care Hospital, Wuhan 430072, China

**Keywords:** Anticholinergic agent, Asthma, Children, Tiotropium

## Abstract

**Objective:**

To investigate the clinical efficacy of tiotropium in children with asthma.

**Methods:**

Eighty children with newly diagnosed moderate persistent asthma were enrolled into this study. The children were randomly assigned to the fluticasone propionate aerosol group or the fluticasone propionate aerosol plus tiotropium group for 12 weeks.

**Results:**

Lung function was significantly improved in both groups at 4, 8, and 12 weeks compared with baseline (*P* < 0.01). Moreover, lung function was significantly improved in the tiotropium group compared with the control group (*P* < 0.05). However, there was no significant difference in the incidence of severe asthma between the two groups (36.3% and 26.8%, respectively; *P* > 0.05). Compared with the control group, the number of days and frequency of short-acting beta2-adrenoceptor agonist use was significantly reduced in the tiotropium group (*P* < 0.05). Awakenings during the night were also significantly decreased (*P* < 0.00). There were no severe adverse reactions in either of the study groups.

**Conclusion:**

Tiotropium could significantly improve lung function, reduce the use of short-acting beta2-adrenoceptor agonists, and improve sleep in children with asthma. Furthermore, few adverse reactions were reported.

## INTRODUCTION

Asthma is a common chronic inflammatory disorder of the airways which is associated with airway hyperresponsiveness, smooth muscle spasm, and airflow obstruction. Over recent years, the global rates of asthma have increased significantly. In USA, about 7 million children and adolescents suffer from asthma,^1^ in the UK, one in every seven children aged 2-15 has asthma symptoms requiring regular treatment^2^ and in China, about 232 in every 10 thousand children under 14 years in the urban has asthma, the rate is more higher than 10 years ago (which is 154 in every 10 thousand).^3^

Inhaled glucocorticoids are considered the first-line treatment for patients with asthma.^4^, ^5^ But many patients taking an inhaled glucocorticoid are inadequately controlled which represents a significant healthcare concern.^6^, ^7^ In combination with inhaled long-acting beta2-adrenoceptor agonists (LABAs) can improve asthma control in children.^8^ However, the long-term use of LABAs is associated with tolerance to the protective effects on the bronchi and an increased risk of fatal asthma exacerbations. The US Food and Drug Administration (FDA) have concerns regarding the safety of LABAs and they recommend that patients discontinue LABAs upon control of asthma symptoms.^9^, ^10^ So alternative medications for bronchospasm relief are required.

Tiotropium is a long-acting anticholinergic bronchodilator^11^ and many researchers consider the effect of tiotropiu is superior to that of LABA,^12^, ^13^ which was effective in COPD.^14^, ^15^ Recently several studies have demonstrated that tritropium is effective in the treatment of asthma in adults^16^, ^17^ and adolescents.^18^ Here we report our data on evaluating the efficacy and safety of tritropium in children. We observed the effects on lung function, exacerbation frequency, awakening during the night, and other end pints during a 12-week period in children with asthma.

## METHODS

Eighty children aged 6-14 years (mean: 10.2±3.6 years) who were diagnosed with moderate persistent asthma in our hospital between December 2013 and December 2014 were enrolled into this study. All the children conformed to the 2008 diagnostic criteria of childhood asthma from the national pediatric asthma association. Eligible children had not received systemic corticosteroids, beta2-adrenoceptor agonists, or cholinergic receptor antagonists for four weeks before enrolment into the study. None of the children had contraindications for corticosteroids use, hypertension, heart disease, or glaucoma. This study was conducted in accordance with the declaration of Helsinki. Approval from the Ethics Committee of Hubei Maternal and Child Health Care Hospital. Written informed consent was obtained from all participants’ guardians. Additionally, the children could use the metered dose inhaler and inhalation system for tiotropium correctly.

The 80 children were randomly assigned to receive either 125 μg fluticasone propionate aerosol twice daily plus placebo once daily (control group), or 125 μg fluticasone propionate aerosol twice daily plus 18 μg tiotropium dry-powder inhaler once daily (treatment group). The study duration was 12 weeks. If the children experienced acute asthma symptoms during the study, short-acting beta2-adrenoceptor agonists, such as 100 μg albuterol aerosol, were permitted as rescue medication. There were no significant differences between the study groups at baseline in age, sex, body mass index (BMI), forced expiratory volume in one second (FEV_1_%), forced vital capacity (FVC), and peak expiratory flow (PEF%) (Table-I). Tiotropium dry-powder capsules (18 μg) were provided by Si Lihua, Boehringer Ingelheim Company, lot number: 301564; fluticasone propionate aerosol (125 μg) was provided by GSK Company, lot number: YJ0225; and albuterol aerosol (100 μg) was provided by Wan Tuolin, GSK Company, lot number: BB0226.

**Table-I:** Comparisons of BMI, age and lung function between two groups (χ̄±s).

Groups	n	Male: Female	BMI	Age	FVC(L)	PEF%	FEV_1_%
Control group	40	24:16	18.2±3.0	10.1±4.8	1.55±0.56	68.57±3.42	67.41±8.51
Treatment group	40	25:15	18.5±3.1	9.3±5.2	1.53±0.47	69.88±3.55	67.31±7.32

### Lung function testing

Lung function testing was performed using a Jaeger MasterScreen pulmonary function testing system (Germany) to examine the tidal breathing flow volume curve at weeks 0 (baseline), 4, 8, & 12. Lung function tests included FEV_1_%, FVC, and PEF.

### Incidence of severe asthma

Severe asthma was defined as an exacerbation that required treatment with oral corticosteroids or hospitalization, or where lung function was reduced by 30% compared to baseline. If severe asthma occurred more than twice within the 12-week study period, patients were withdrawn from the study. The frequency of use and number of days of use of short-acting beta2-adrenoceptor agonists were recorded. Night-time awakenings were also recorded.

### Statistical analysis

Statistical analysis was performed using SPSS 10.0 software. The results are presented as mean±SD. A paired t-test was used to analyze count data, and a chi-square test was used to analyze between-group probability. A p-value of <0.05 was considered statistically significant.

### Inclusion Criteria

1. Newly diagnosed moderate persistent asthma; 2. Can use the HandiHaler properly; 3. Without use of any glucocorticoid within 4 weeks and without use of any beta2-adrenoceptor agonists within one weeks; 4. No hormone contraindications.

### Exclusion Criteria

Those who has heart disease, lung infection disease, glaucoma or other complications.

## RESULTS

Two children in each of the study groups were withdrawn from the study following >2 severe asthma exacerbations. Therefore, four cases were not included in the statistical analysis. As shown in Table-I, there were no significant differences for BMI, age, and lung function between the 2 groups (*P* > 0.05).

The FEV_1_%, FVC, and PEF% of patients in both groups were significantly improved at weeks 4, 8, and 12 compared to baseline (*P* < 0.01; Table-II). Moreover, the improvement in lung function in the treatment group was significantly greater than in the control group (FEV_1_% at week 12 and FVC at week 8 *P* < 0.05, all other indicators *P* < 0.01).

**Table-II:** Changes of FEV1%, FVC and PEF% before and after treatment in two groups (χ̄±s).

*Groups*	*Case*	*FEV_1_%*			*FVC(L)*			*PEF%*		

		*4^th^ week*	*8^th^ week*	*12^th^ week*	*4^th^ week*	*8^th^ week*	*12^th^ week*	*4^th^ week*	*8^th^ week*	*12^th^ week*
Control	38	73.91±5.66 ^▲^	82.34±5.02 ^▲^	87.53±5.76 ^▲^	1.63±0.47 ^▲^	1.70±0.51 ^▲^	1.80±0.59 ^▲^	77.39±3.56 ^▲^	81.63±4.76 ^▲^	86.35±3.65 ^▲^
Treatment	38	79.56±7.01 ^▲#^	87.44±6.74 ^▲#^	91.61±5.73 ^▲*^	1.87±0.42 ^▲*^	1.95±0.48 ^▲*^	2.42±0.55 ^▲#^	80.0±3.68 ^▲#^	85.52±3.9 ^▲#^	89.67±4.14 ^▲#^

▲ vs before the treatment *P* < 0.01;

* vs. control group *P* < 0.05,

# *P* < 0.01

The incidence of severe asthma was 36.8% (n = 14/38) in the control group and 26.3% (n = 10/38) in the treatment group, but the difference between the two groups was not significant (χ^2^ = 0.54, *P* > 0.05). Two patients who presented with severe asthma in each of the study groups were withdrawn from the study.

The frequency and number of days of use of on-demand short-acting beta2-adrenoceptor agonists were significantly reduced in the treatment group compared to the control group (*P* < 0.05; Table-III). Furthermore, significantly fewer night-time awakenings were reported in the treatment group compared to the control group (*P* < 0.00).

**Table-III:** Comparisons of frequency and days of use of short acting beta 2 receptor agonist on demand and awakening days during the night (χ̄±s).

Groups	Cases	Frequency	Days	Awakening days during the night
Control group	38	0.65±0.34	42.1±16.5	45.1±10.6
Treatment group	38	0.51±0.23*	32.9±14.3*	28.4±6.9**

Vs. control group

* P < 0.05,

** P < 0.0.

### Adverse reactions

In the treatment group, there were three reports of adverse reactions. All three patients presented with xerostomia and throat discomfort; however, the symptoms were relieved when the children were given more water and instructed to gargle water after administration of the medication. There were 2 adverse reactions reported in the control group. One patient presented with hoarseness, and another presented with thrush, which was successfully treated with myclin and oral nursing. There were no reports of hypertension, tachycardia, or glaucoma in either of the study groups.

## DISCUSSION

FEV1%, FVC and PEF% are the standard parameter of lung functions. Our results show that, inhaled corticosteroids can improve the lung function of both the control and treatment groups, the result was the same with Pauwels.^19^ When added with tiotropium the lung function of treatment group was improved better than placebo. The result should be connected with tiotropium’s subtype selectivity.^20^ Vogelberg’s nearest study had demonstrated similar effects on children, although the end point was not the same with us.^21^

Awakening during the night is an important factor that affects quality of life in all patients with asthma, including children. A large-scale investigation by Warvik^22^ found that 39% of patients with asthma were woken every night due to symptoms, and 74% of patients reported awakening at night at least once a week. Furthermore, 70% of deaths due to asthma happen at night during sleep. Our study found that the tiotropium can reduce the frequency and the days of using the short-acting beta2-adrenoceptor agonists and also reduce the awakening days during the night. The reason may be that the parasympathetic nervous system is an important mechanism in nocturnal asthma symptoms^23^ and the long half-life of tiotropium which is nearly 35 hours and the efficacy can last for a whole day.^24^ Vogelberg’s research had not mentioned it.^21^

Some studies show that the addition of tiotropium can significantly reduced the risk of episodes of the worsening. Our study showed the incidence of severe asthma was lower in the treatment group than in the control group, but the difference between the two groups was not significant. Therefore, a beneficial effect of tiotropium on the risk of severe asthma could not be proved. We think there may be other causal factors of severe asthma exacerbations, for example, allergic rhinitis and nasosinusitis, can complicate treatment.^25^

In conclusion, tiotropium, a long-acting cholinergic receptor antagonist, could significantly improve lung function, reduce the need for on-demand short-acting beta2-adrenoceptor agonists, and improve nocturnal symptoms in children with asthma. Furthermore, tiotropium was associated with few adverse reactions in this patient group. These advantages indicate that tiotropium could be a promising prospect for the future for the treatment of asthma in children.
